# Communication Education in Prelicensure and Postlicensure Occupational Therapy: Scoping Review

**DOI:** 10.2196/95909

**Published:** 2026-11-13

**Authors:** Ryuichi Shimada, Yukiko Sano, Takeshi Sasaki, Astuko Tanimura

**Affiliations:** 1 Department of Occupational Therapy, Faculty of Health Sciences, Iryo Sosei University, 5-5-1 Chuodai Iino, Iwaki, Fukushima, 970-8551, Japan, 81 246-88-6475; 2 Department of Occupational Therapy, Graduate School of Human Health Sciences, Tokyo Metropolitan University, Arakawa-ku, Tokyo, Japan; 3 Comuro Co, Ltd, Visiting Nursing Station Ai, Fukushima, Fukushima, Japan

**Keywords:** occupational therapy, communication, education, communication skills, scoping review

## Abstract

**Background:**

Effective communication, therapeutic relationships, and interprofessional collaboration are essential to high-quality occupational therapy (OT) practice. However, communication education across prelicensure and postlicensure OT training has not been comprehensively mapped.

**Objective:**

This review aims to identify research gaps and map communication education in prelicensure and postlicensure OT education according to content, delivery methods, assessment approaches, and outcomes.

**Methods:**

Following Joanna Briggs Institute guidance, this review was reported using the Preferred Reporting Items for Systematic Reviews and Meta-Analyses extension for Scoping Reviews (PRISMA-ScR) and Preferred Reporting Items for Systematic Reviews and Meta-Analyses Literature Search Extension (PRISMA-S) frameworks. PubMed, CINAHL, ERIC, Web of Science, Ichushi-Web, and Open Access Theses and Dissertations were searched without publication year restrictions; the final search was conducted on May 25, 2026. Eligible sources were English- or Japanese-language publications that examined communication education involving OT students or practicing occupational therapists. The review protocol was developed a priori but was not registered. Two reviewers independently screened records identified through database and gray literature searches, whereas records identified through backward citation searching were screened by one reviewer and checked by the second reviewer. Data were charted for study characteristics, educational content, delivery methods, assessment approaches, and outcomes. Educational outcomes were categorized using the Kirkpatrick model. Formal critical appraisal was not conducted, and the findings were synthesized descriptively.

**Results:**

Database and gray literature searches identified 10,243 records, and backward citation searching identified 9 additional records. After duplicate removal and screening, 55 reports corresponding to 54 studies were included. Of the 55 reports, 47 (85.5%) focused on prelicensure education and 8 (14.5%) on postlicensure education; prelicensure reports mainly involved OT students learning either within OT programs alone or with students from other health professions. Single-group pre-post studies were the most common design (n=22, 40%). Educational content was diverse and included interprofessional communication and teamwork, communication with specific client populations or conditions, model-based communication training, experiential communication skills training, and OT-specific therapeutic communication or therapeutic use of self. Outcome assessment relied mainly on learner-reported measures, including perceived competence, self-efficacy, self-reported communication skills, and attitudes. Objective or observer-rated assessments were less common. Across the 55 included reports, 15 (27.3%) reported reaction-level outcomes, 53 (96.4%) learning-level outcomes, and 5 (9.1%) behavior-level outcomes; no report assessed results-level outcomes, and reports could contribute to more than one level.

**Conclusions:**

This review maps communication education across both prelicensure and postlicensure OT education in the English- and Japanese-language literature. The findings highlight the need for more explicit occupation-centered education, including occupational history taking, values-based goal setting, therapeutic relationship development, and therapeutic use of self. They may inform curriculum design, continuing professional development, and outcome measure selection. Interpretation should remain limited to the included literature, which was dominated by single-group pre-post designs and learner-reported outcomes.

## Introduction

High-quality occupational therapy (OT) depends on effective communication with clients, their families, and other professionals. The primary goal of OT is to support client participation in meaningful daily activities, thereby facilitating role and routine restoration and improving quality of life [1]. Achieving this goal requires occupational therapists to understand clients’ values and life histories through client-centered communication and to integrate this information into goal setting and intervention planning. Therapeutic alliances grounded in empathic understanding [2], as well as effective interprofessional collaboration [3], are associated with improved clinical outcomes. Communication functions as a fundamental clinical tool in OT for promoting occupational participation. Therefore, OT education must develop essential professional competencies, including (1) eliciting occupational histories that reflect clients’ values and life experiences [1], and (2) intentionally applying therapists’ attitudes and engagement strategies to establish and sustain therapeutic relationships and support intervention (therapeutic use of self) [4]. The World Federation of Occupational Therapists (WFOT) Minimum Standards for the Education of Occupational Therapists 2026 identify elements of therapeutic interaction and communication skills, team-based learning and collaboration, and complex clinical reasoning as competencies for which online methods should be supplemented by in-person methods [5]. Although these standards define common competencies for accredited entry-level programs worldwide, they do not prescribe how communication education should be designed, delivered, or assessed. Curricula and teaching methods are shaped by national regulatory frameworks, entry-level degree structures, available educational resources, and cultural and linguistic contexts, which may contribute to variation in how communication education is implemented and reported across countries and settings. In Japan, the “Model Core Curriculum for Occupational Therapist Education” identifies communication competence, including relationship development through active listening and empathy, as a core competency [6]. Together, these international and national frameworks support the need to examine how communication education has been delivered and evaluated in OT education across different educational stages and contexts.

Within OT, theory-informed communication education has been described, including approaches based on the intentional relationship model, which conceptualizes the therapeutic use of self [4]. Recent qualitative work has further described therapeutic use of self as a relational pedagogy in OT education, reflecting growing attention to how communication and therapeutic relationships are taught [7]. However, substantial heterogeneity in educational methods and outcome measures has limited synthesis across studies. Furthermore, outcomes frequently emphasize subjective or indirect indicators, such as self-efficacy and knowledge, rather than observable communication behaviors or client outcomes. Consequently, evidence linking communication education to measurable clinical communication performance and client outcomes remains limited. Previous reviews in other health professions have reported that communication skills training and interprofessional education are associated with improvements in learner-reported outcomes and collaborative practice-related outcomes [8-10]. In OT education, related reviews have examined simulation-based education and coaching [11,12]. However, these bodies of literature do not specifically map how communication education in OT has been designed, delivered, and evaluated across both prelicensure and postlicensure education.

In this review, communication education refers to educational activities designed to develop occupational therapists’ competencies in communicating with clients, families, and other professionals across prelicensure and postlicensure education. It includes education related to therapeutic relationships, eliciting clients’ occupational histories and values, collaborative goal setting, communication with specific client populations or conditions, and interprofessional collaboration. Accordingly, this scoping review aims to map the content, delivery methods, assessment approaches, and outcomes of communication education in prelicensure and postlicensure OT education and to identify gaps in the existing literature. The review questions were as follows: (1) What study characteristics, educational content, and delivery methods have been reported for communication education in prelicensure and postlicensure OT education? and (2) What assessment approaches and outcomes have been reported?

## Methods

### Scoping Review Framework and Reporting Guidelines

A scoping review methodology was used to map relevant literature and summarize existing evidence. This approach is appropriate for examining the scope and characteristics of evidence within a field and identifying research gaps, consistent with the objectives of this study [13]. The methodology followed Joanna Briggs Institute guidance [14], and the findings were reported according to the Preferred Reporting Items for Systematic Reviews and Meta-Analyses extension for Scoping Reviews (PRISMA-ScR) framework [15]. The search strategy was reported with reference to the Preferred Reporting Items for Systematic Reviews and Meta-Analyses Literature Search Extension (PRISMA-S) framework [16]. The PRISMA-ScR checklist is included as Checklist 1. Formal critical appraisal of individual sources of evidence, including assessment of methodological quality or risk of bias, was not conducted.

### Protocol and Registration

Because communication is a broad and multifaceted concept and is described using diverse terminology in the OT literature, preliminary searches were conducted to clarify relevant terminology and refine the eligibility criteria. A protocol was developed before the formal search and screening as an internal working document. It was not prospectively registered or publicly posted at project initiation. During manuscript revision, the internal protocol was retrospectively archived on the Open Science Framework [17]. The search strategy was subsequently expanded and updated from the protocol by adding education-focused and multidisciplinary databases and a gray literature source. No other major deviations from the a priori protocol occurred.

### Eligibility Criteria

Eligibility criteria were formulated using the population-concept-context framework. The “population” comprised OT students and practicing occupational therapists, reflecting both prelicensure and postlicensure education. The “concept” was communication education, as defined in the Introduction section. Studies were eligible when communication was explicitly addressed as an educational objective, educational content, or outcome assessment. Eligible communication topics included, but were not limited to, interpersonal communication, therapeutic relationships, therapeutic use of self, interviewing, shared decision-making, motivational interviewing, patient- or client-centered communication, collaborative goal setting, communication with specific client populations or conditions, and interprofessional communication or teamwork. The “context” included English- and Japanese-language publications without publication year restrictions.

The inclusion criteria were as follows: (1) the studies examined an educational activity related to communication; (2) the participants included OT students or occupational therapists; (3) communication-related elements were explicitly described as an educational objective, educational content, or outcome assessment; and (4) communication-related data were sufficiently extractable for data charting. Studies involving interprofessional education or participants from multiple disciplines were included only when OT students or occupational therapists were included, and communication-related data relevant to the review questions could be extracted. Sources were excluded if they met any of the following criteria: (1) they did not examine communication education; (2) communication-related educational components or outcome data were not sufficiently distinguishable or extractable; (3) they did not include OT students or occupational therapists; (4) they focused only on the development or validation of an assessment measure; (5) they were review papers; or (6) they were books, conference abstracts, editorials, commentaries, or case studies. In addition, studies in which communication was only incidental to the educational activity and was not described as an educational objective, educational content, or outcome assessment were excluded.

### Information Sources

PubMed (MEDLINE), CINAHL, ERIC, Web of Science, and Ichushi-Web (Japan Medical Abstracts Society) were searched. Open Access Theses and Dissertations (OATD) was also searched as a gray literature source. These sources were selected to cover biomedical and allied health literature (PubMed, CINAHL), multidisciplinary literature (Web of Science), education-focused literature (ERIC), and Japanese-language literature (Ichushi-Web), with OATD capturing gray literature in the form of theses and dissertations. Controlled vocabulary terms, where available, and free-text terms were combined and adapted for each database. No publication year restrictions were applied. The final search was conducted on May 25, 2026.

In addition to database searching, backward citation searching was conducted by manually screening the reference lists of included reports to identify additional potentially relevant sources. Forward citation searching was not conducted because it was not prespecified in the review protocol and could not be applied consistently to all included reports within the review timeline. Study registries were not searched, and authors or experts were not contacted to identify additional sources. The search strategy was developed and refined through discussion within the research team; however, formal peer review using the Peer Review of Electronic Search Strategies (PRESS) 2015 guideline was not conducted.

### Search

Search strategies were developed using the population-concept-context framework. The search covered 3 core concepts: OT, communication, and education. Controlled vocabulary terms, such as MeSH terms, were used where available, and free-text terms were searched using database-specific field modifiers and syntax. The search strategy included terms related to OT, such as “occupational therapy” and “occupational therapist”; communication-related terms, such as “communication,” “professional-patient relations,” “interprofessional relations,” “interview,” “therapeutic relationship,” “therapeutic use of self,” “patient-centered communication,” and “interprofessional communication”; and education-related terms, such as “education,” “training,” “curriculum,” “learning,” and “competency.” No publication year filters were applied. The full search strategies for each database and information source, including database-specific syntax, limits, filters, search dates, and records retrieved, are provided in Multimedia Appendix 1.

### Selection of Sources of Evidence

Retrieved records were compiled in Microsoft Excel, and duplicate records were removed by comparing bibliographic information, including titles, authors, publication years, and journal names. Two reviewers (the first and second authors) independently screened records identified through the bibliographic database searches and the OATD gray literature search, with disagreements resolved through discussion and consensus. Records identified through backward citation searching were screened by one reviewer and subsequently checked by the second reviewer; these records were not independently screened in duplicate. Interreviewer agreement before consensus was assessed separately at the title and abstract and full-text screening stages using percentage agreement and unweighted Cohen κ with 95% CIs, calculated using the vcd package (version 1.4‐14) in R (version 4.6.1; R Foundation for Statistical Computing). The selection process was conducted in two stages. First, titles and abstracts were screened to identify potentially eligible sources. Second, full texts were assessed against the eligibility and exclusion criteria. For sources involving interprofessional education or broader educational programs, eligibility was determined by whether communication-related educational components or outcomes relevant to OT students or occupational therapists could be clearly identified and extracted.

### Data Charting Process

A data charting form was developed in Microsoft Excel based on the review questions and eligibility criteria. The form was refined through initial extraction and team discussion to ensure that the charted items aligned with the review questions. One reviewer (RS) extracted data from all included reports. Two additional reviewers (YS and TS) reviewed the completed data charting spreadsheet and summary tables and checked the corresponding original reports when clarification or verification was needed. Data were not independently extracted in duplicate. Discrepancies or questions were resolved through discussion and consensus. When reports involved interprofessional education or broader educational programs, the reviewers charted only communication-related educational components and outcomes that were relevant to the review questions.

### Data Items

The charted data items included bibliographic information, country, publication year, study design, participant characteristics, education stage, educational aims, educational content and delivery methods, communication-related assessment approaches, communication-related outcome measures, and communication-related findings. Educational content categories and Kirkpatrick outcome levels were subsequently assigned during synthesis.

### Critical Appraisal of Individual Sources of Evidence

Formal critical appraisal of individual sources of evidence was not conducted. This approach was consistent with the purpose of this scoping review, which was to map the extent, characteristics, and gaps in the published literature rather than to determine intervention effectiveness or the certainty of evidence. Reported outcomes were therefore interpreted descriptively and were not used to infer intervention effectiveness.

### Synthesis of Results

Extracted data were summarized using descriptive statistics. Study designs were categorized as randomized controlled trials, nonrandomized controlled trials, single-group pre-post studies, mixed methods studies, qualitative studies, or practice reports/descriptive studies. Educational content categories were developed inductively from the charted data. Educational content and outcomes were initially coded by one reviewer and subsequently reviewed by the research team. Because independent duplicate coding was not conducted, interrater reliability statistics were not calculated. Categories were refined through discussion until consensus was reached. Outcome measures were categorized according to the primary information source or assessment approach into 3 categories: self-report measures, qualitative data sources, and objective or observer-rated assessments. Qualitative data sources included qualitative text analysis, open-ended questionnaires, interviews or focus groups, and qualitative analysis of video-recorded data. Educational outcomes were also classified using the Kirkpatrick model: level 1, reaction; level 2, learning; level 3, behavior; and level 4, results [18]. This model was used to classify outcomes from learners’ perceptions and knowledge acquisition to behavioral change and client- or practice-level results. Analyses were conducted at the report level, covering 55 reports. Each report was assigned to a mutually exclusive study design category and one primary educational content category. A report could contribute to more than one Kirkpatrick outcome level when multiple levels were assessed, but it was counted only once within each level. We descriptively cross-tabulated educational content and study design by Kirkpatrick level and visualized these distributions as an evidence gap map and a heatmap; no inferential comparisons were performed. Both visualizations were generated using R (version 4.6.1).

## Results

### Selection of Sources of Evidence

Database searches and gray literature yielded 10,243 records. After removing duplicates, 6562 records underwent title and abstract screening, resulting in the exclusion of 6381 records. Full texts were sought for 181 reports, of which 19 (10.5%) could not be retrieved. A total of 162 reports were assessed for eligibility, and 110 reports were excluded, yielding 52 reports from the database searches. At the title and abstract screening stage, the 2 reviewers agreed on 6419 of 6562 (97.8%) database-derived records, with an unweighted Cohen κ of 0.610 (95% CI 0.551‐0.669). At the full-text screening stage, they agreed on 137 of 162 (84.6%) database-derived reports, with an unweighted Cohen κ of 0.601 (95% CI 0.461‐0.742). Exclusion decisions predominated for both reviewers at the title and abstract screening stage (reviewer 1: 6412/6562, 97.7%; reviewer 2: 6335/6562, 96.5%) and the full-text screening stage (reviewer 1: 120/162, 74.1%; reviewer 2: 119/162, 73.5%). The corresponding expected chance agreement was 94.4% and 61.3%, respectively. All disagreements were resolved through discussion and consensus. Backward citation searching of the reference lists of included reports identified 9 additional records; all 9 full-text reports were retrieved and assessed for eligibility, of which 6 were excluded and 3 were included. Two included reports were based on the same study cohort, with one report examining longer-term outcomes in the same participants (Multimedia Appendix 2). Consequently, 55 reports corresponding to 54 studies were included in the final analysis (Figure 1). Additional details of the included studies are provided in Multimedia Appendix 2.

**Figure 1.**
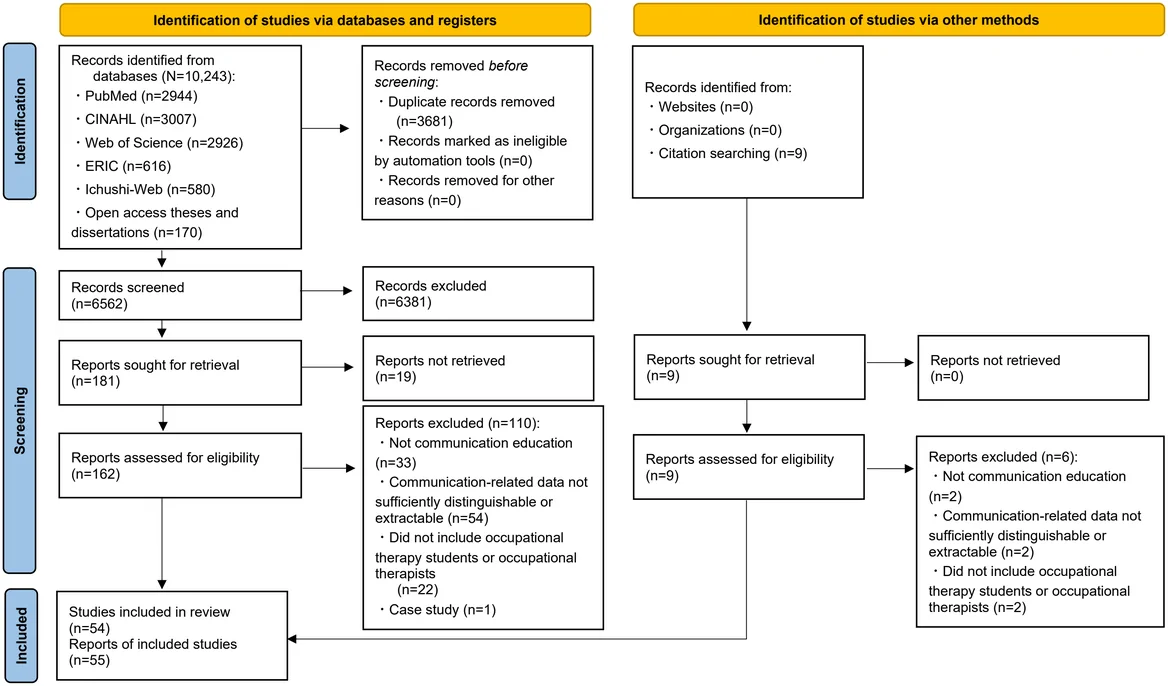
PRISMA (Preferred Reporting Items for Systematic Reviews and Meta-Analyses) 2020 flow diagram for the identification and selection of reports on communication education in prelicensure and postlicensure occupational therapy, with searches conducted through May 25, 2026. The flow diagram was adapted from the PRISMA 2020 for new systematic reviews. No automation tools were used to exclude records. ERIC: education-focused literature.

### Characteristics of Sources of Evidence

The 54 included studies were represented by 55 reports. Table 1 summarizes the characteristics of the included reports. Most reports were published in 2020 or later (n=29, 52.7%) or between 2010 and 2019 (n=24, 43.6%). The largest number of reports came from the United States (n=22, 40%), followed by Australia (n=13, 23.6%). Most reports focused on prelicensure education. Studies involving OT students and students from other health professions were most common (n=28, 50.9%), followed by studies involving only OT students (n=20, 36.4%). Reports involving practicing occupational therapists were limited (n=7, 12.7%), including 2 reports involving only occupational therapists (n=2, 3.6%) and 5 involving occupational therapists and other professionals (n=5, 9.1%). Single-group pre-post studies were the most frequent design (n=22, 40.0%), followed by mixed methods studies (n=15, 27.3%). Controlled intervention studies accounted for 7 reports, including randomized controlled trials (n=5, 9.1%) and nonrandomized controlled trials (n=2, 3.6%).

**Table 1.** Characteristics of the 55 included reports (54 studies) on communication education in prelicensure and postlicensure occupational therapy (OT) education, 1997‐2026^a^.

Characteristics and category	Reports, n (%)
Publication year	
1997‐2009	2 (3.6)
2010‐2019	24 (43.6)
2020‐2026	29 (52.7)
Country	
United States	22 (40)
Australia	13 (23.6)
Japan	6 (10.9)
United Kingdom	4 (7.3)
Taiwan	3 (5.5)
Norway	2 (3.6)
Canada	1 (1.8)
China	1 (1.8)
Ireland	1 (1.8)
Israel	1 (1.8)
Sweden	1 (1.8)
Participant group	
OT students only	20 (36.4)
OT students and other health profession students	28 (50.9)
Occupational therapists only	2 (3.6)
Occupational therapists and other professionals	5 (9.1)
Study design	
Single-group pre-post study	22 (40)
Mixed methods study	15 (27.3)
Descriptive survey or practice report	6 (10.9)
Randomized controlled trial	5 (9.1)
Qualitative study	5 (9.1)
Nonrandomized controlled trial	2 (3.6)

a Percentages were calculated using all included 55 reports as the denominator. One study (Country column) was represented by 2 reports; therefore, counts are reported at the report level.

### Outcome Measures Used to Evaluate Communication Education

Table 2 summarizes the assessment approaches used to evaluate communication education. Overall, 106 assessment items were extracted. Self-report measures were the most frequently used approach, accounting for 53 (50%) items and primarily assessing perceived competence, confidence, self-efficacy, communication skills, and attitudes. Qualitative data sources accounted for 29 (27.4%) items and included open-ended questionnaires, focus groups, and interviews exploring learners’ experiences and perceived changes after education. Objective or observer-rated assessments accounted for 24 (22.6%) items and included knowledge tests, objective structured clinical examinations, rubric-based ratings, video coding, and external ratings. Overall, evaluations of communication education were more often based on learner-reported outcomes than on objective, observer-rated, or performance-based assessments.

**Table 2.** Assessment approaches used across 55 reports representing 54 studies of communication education in prelicensure and postlicensure occupational therapy, 1997‐2026 (N=106)^a^.

Assessment approach	Description	Examples	Items, n (%)
Self-report measures	Learner-reported scales or questionnaires assessing perceived competence, confidence, or self-efficacy	SETUS^b^, ICCAS-R^c^, and original questionnaires	53 (50)
Qualitative data sources	Interview or text-based data used to explore learner experience, perceived changes after education	Open-ended questionnaires and focus groups	29 (27.4)
Objective or observer-rated assessments	Structured assessments of knowledge or communication performance rated by faculty, standardized patients, or external raters	Knowledge tests, OSCE^d^, rubric-based ratings, video coding, and MITI^e^	24 (22.6)

a Counts represent assessment items, not reports. Each assessment item was assigned to one primary assessment approach. A single report could include multiple assessment items and could contribute to more than one assessment category. Percentages were calculated using the total number of extracted assessment items as the denominator (N=106).

b SETUS: self-efficacy for therapeutic use of self.

c ICCAS-R: Interprofessional collaborative competencies attainment survey (revised).

d OSCE: objective structured clinical examination.

e MITI: motivational interviewing treatment integrity.

### Content, Delivery Methods, and Outcomes of Communication Education

#### Educational Content and Delivery Methods

Table 3 summarizes the educational content categories by education stage. Across the 55 reports, most reports focused on prelicensure education (n=47, 85.5%), whereas postlicensure education was reported less frequently (n=8, 14.5%). The most common categories were communication with specific client populations or conditions (n=14, 25.5%) [19-32] and interprofessional communication and teamwork (n=14, 25.5%) [33-46]. Communication with specific client populations or conditions included education focused on communication with people with aphasia, dementia, communication disorders, or cancer. Interprofessional communication and teamwork were limited to prelicensure education and included interprofessional education simulations, interprofessional cotreatment, and virtual patient learning. Technique- or model-based communication training was reported in 9 (16.4%) reports [47-55] and included approaches such as motivational interviewing, shared decision-making, and client-centered counseling. General experiential communication skills training was reported in 10 (18.2%) reports [56-65] and included broad communication skills training and real-patient conversation practice. OT-specific therapeutic communication and therapeutic use of self was reported in 8 (14.5%) reports [66-73], including education grounded in therapeutic use of self and therapeutic relationship development. Delivery methods varied across reports and included lectures, simulation-based learning, role-play or standardized patient encounters, real-patient encounters, discussions, reflective activities, and online or virtual learning. These methods were often combined within the same educational program. Details for each report are provided in Multimedia Appendix 2.

**Table 3.** Educational content categories across 55 reports representing 54 studies of communication education in prelicensure and postlicensure occupational therapy (OT), 1997‐2026^a^.

Educational content category	Definition	Examples	Prelicensure, n (%)	Postlicensure, n (%)	Total reports, n (%)
OT-specific therapeutic communication and therapeutic use of self	Education grounded in OT-specific concepts of therapeutic communication	IRM^b^ or therapeutic use of self [66-73]	7 (12.7)	1 (1.8)	8 (14.5)
Communication with specific client populations or conditions	Education focused on communication strategies for specific client groups or conditions	Communication with people with aphasia, dementia, communication disorders, and cancer [19-32]	9 (16.4)	5 (9.1)	14 (25.5)
Interprofessional communication and teamwork	Education addressing communication within interprofessional collaboration	IPE^c^ simulation and interprofessional cotreatment [33-46]	14 (25.5)	0 (0)	14 (25.5)
Technique- or model-based communication training	Education based on a named communication approach or model	Motivational interviewing and shared decision-making [47-55]	7 (12.7)	2 (3.6)	9 (16.4)
General experiential communication skills training	Broad experiential learning aimed at general communication	Communication skills training and real-patient conversation practice [56-65]	10 (18.2)	0 (0)	10 (18.2)
Total			47 (85.5)	8 (14.5)	55 (100)

a Percentages were calculated using all included reports as the denominator (N=55). Each report was assigned to one primary educational content category. Reference numbers in brackets indicate the reports in each category.

b IRM: intentional relationship model.

c IPE: interprofessional education.

#### Outcomes of Communication Education

Across the 55 reports, 53 (96.4%) reported level 2 (learning) outcomes, 15 (27.3%) level 1 (reaction) outcomes, 5 (9.1%) level 3 (behavior) outcomes, and none reported level 4 (results) outcomes. Percentages do not sum to 100% because reports could contribute to more than one Kirkpatrick level.

Figure 2 presents an evidence gap map of educational content categories and Kirkpatrick outcome levels. Learning-level outcomes were the most frequently reported across all educational content categories. These outcomes included changes in knowledge, confidence, self-efficacy, perceived communication skills, attitudes, and communication-related understanding. Reaction-level outcomes were reported less frequently and were mainly identified in general experiential communication skills training, interprofessional communication and teamwork, and communication with specific client populations or conditions. Behavior-level outcomes were limited. They were identified in technique- or model-based communication training, communication with specific client populations or conditions, interprofessional communication and teamwork, and general experiential communication skills training. None of the reports included in this review assessed results-level outcomes, such as client-level outcomes, occupational participation, quality of life, or broader practice-level effects. Overall, within the reports included in this review, communication education outcomes were concentrated at the learning level, with relatively limited evidence on behavioral change and no reports on results-level outcomes.

Figure 3 presents a heatmap of study designs and Kirkpatrick outcome levels. Learning-level outcomes were represented across all study designs and were most frequently reported in single-group pre-post studies (21 reports) and mixed methods studies (15 reports). Reaction-level outcomes were most frequent in descriptive/practice reports (6 reports) and were not reported in randomized or nonrandomized controlled trials. Behavior-level outcomes were reported in only 5 reports: 3 single-group pre-post studies, 1 mixed methods study, and 1 nonrandomized controlled trial. Behavior-level outcomes were not reported in randomized controlled trials, qualitative studies, or descriptive/practice reports, and no reports in any study design category assessed results-level outcomes.

**Figure 2.**
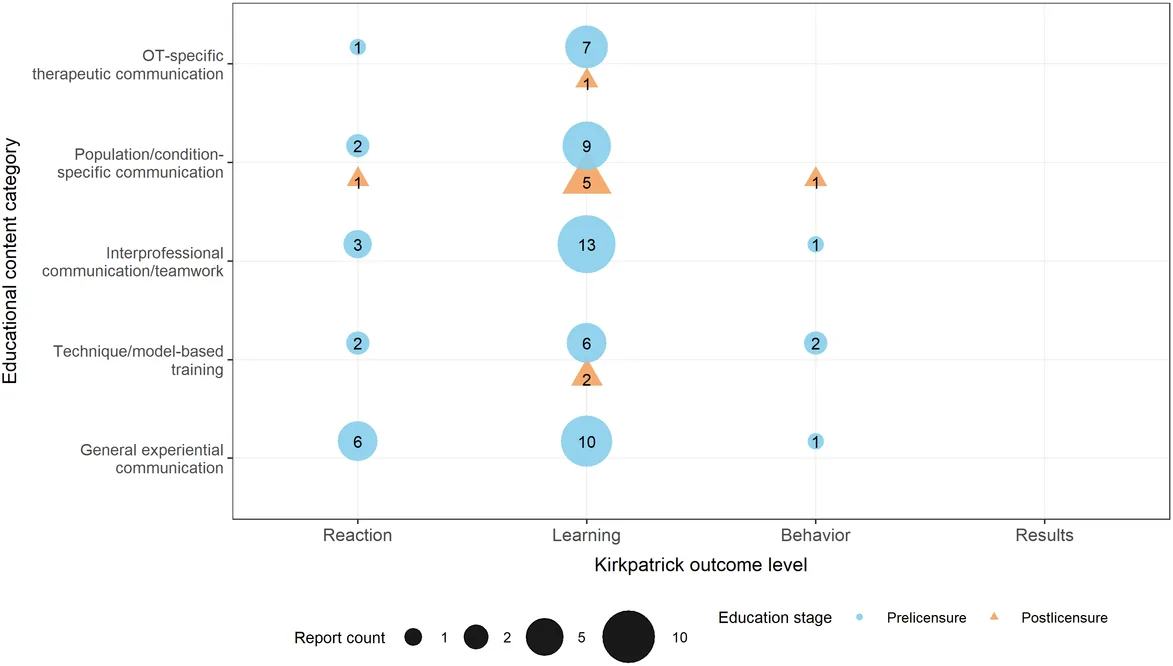
Evidence gap map of educational content categories and Kirkpatrick outcome levels across 55 reports of prelicensure and postlicensure OT communication education, 1997‐2026. Bubble sizes are scaled to the number of reports, as indicated by the size legend (1, 2, 5, and 10 reports); blank cells indicate zero reports. Circles indicate prelicensure education, and triangles indicate postlicensure education. Kirkpatrick level 1=reaction; level 2=learning; level 3=behavior; and level 4=results. None of the included reports assessed level 4 results-level outcomes. Each report was assigned to one primary educational content category; reports could contribute to more than one Kirkpatrick outcome level when multiple outcomes were assessed. Category labels were shortened for readability; full category names are provided in Table 3. OT: occupational therapy.

**Figure 3.**
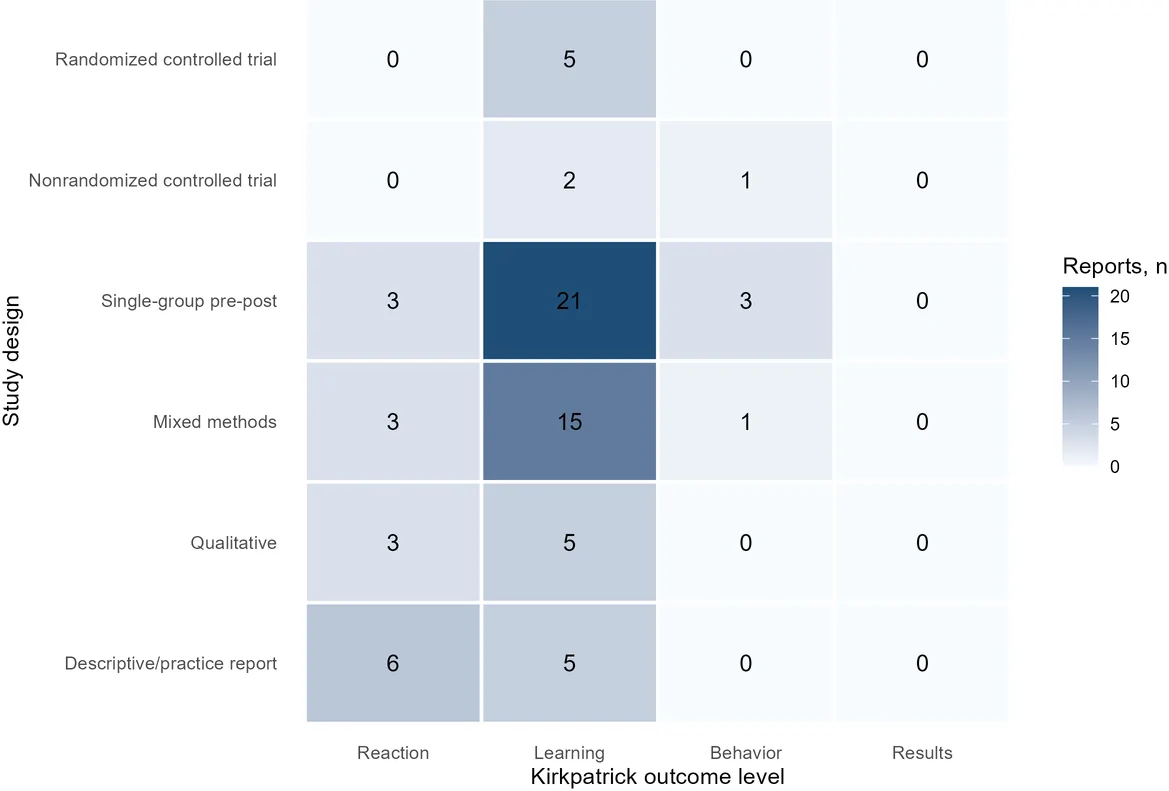
Heatmap of study designs and Kirkpatrick outcome levels across 55 reports of communication education in prelicensure and postlicensure occupational therapy, 1997‐2026. Cell values and color intensity indicate the number of reports. Reports could contribute to more than one Kirkpatrick outcome level when multiple outcomes were assessed. Kirkpatrick level 1=reaction; level 2=learning; level 3=behavior; and level 4=results.

## Discussion

### Summary of Evidence

The included reports were concentrated mainly in prelicensure education and addressed diverse educational content and delivery methods. Outcome assessment relied primarily on learner-reported measures, whereas objective or observer-rated assessments were less common. Within the reports included in this review, Kirkpatrick outcomes were concentrated at the learning level, behavior-level outcomes were limited, and results-level outcomes were not assessed. A similar pattern was observed across study designs, with none of the randomized controlled trials assessing behavior- or results-level outcomes. Overall, these patterns highlight the limited evaluation of observable communication behavior and client- or practice-level outcomes.

### Comparison With Existing Literature

The concentration of reports in prelicensure education is consistent with broader trends in health professions education, where communication skills, teamwork, and simulation-based learning are commonly introduced during professional preparation [8,10,11]. Previous reviews have reported improvements in learner-reported outcomes, including self-efficacy and confidence, after communication skills training [8], and have shown that simulation-based education is widely used in OT education [11]. The present review extends this literature by showing that, within OT, communication education has often been reported in interprofessional or condition-specific contexts rather than as a distinct occupation-centered educational domain. The prominence of interprofessional communication and teamwork also aligns with the collaborative nature of health and social care practice [3,10]. Interprofessional education has been examined as a strategy to prepare students and practitioners for collaborative practice [10]. In the included OT literature, communication was often embedded within teamwork, role clarification, and other collaborative learning activities. This positioning may provide meaningful opportunities for communication learning [10,11]. However, because communication was often embedded within broader educational activities in the included reports, OT-specific communication tasks may have been less visible.

The findings also correspond with OT literature emphasizing therapeutic relationships and therapeutic use of self as core components of practice [4,74]. These perspectives suggest that OT communication involves not only information exchange but also efforts to understand clients’ values, life histories, occupational identities, and participation goals [1,4,74]. However, the included literature did not consistently make these occupation-centered communication processes explicit as educational targets or assessment outcomes. Alongside this limited specification of occupation-centered communication, the outcome patterns identified in this review are consistent with concerns in the wider communication education literature [75-77]. Previous reviews have noted limitations in outcome measurement, including reliance on learner-reported outcomes and limited assessment of behavioral transfer or client-level outcomes [75-77]. Similarly, among the reports included in this review, learning-level outcomes predominated, behavior-level outcomes were uncommon, and no results-level outcomes were reported. These findings caution against assuming that improvements in learner-reported outcomes translate directly into observable clinical performance or benefits for clients and practice [75-77]. The diversity of educational content and delivery methods identified in this review may appear inconsistent with the existence of entry-level OT programs guided by the WFOT minimum standards [5]. However, the WFOT standards define competencies to be achieved without standardizing specific teaching methods [5]. Programs may therefore adapt communication education to their regulatory, institutional, cultural, and linguistic contexts. In addition, communication education was often embedded within interprofessional or condition-specific learning activities rather than delivered as a distinct curricular unit, and reporting practices may differ across countries and journals. These explanations are hypotheses derived from the mapped literature and warrant comparative research on how communication education is structured across accredited programs.

### Implications for OT Education and Practice

The findings suggest that communication may need to be made more explicit as a learning objective in OT education and in reports of educational interventions. Communication education was often embedded in interprofessional education, condition-specific training, and experiential learning activities such as simulation, role-play, standardized patient encounters, real-patient encounters, discussion, and reflection. These approaches may provide useful opportunities for experiential learning [10,11]. Their educational value may be enhanced when communication learning is grounded in OT-specific clinical situations and occupation-centered practice [1,4]. For OT, communication education may extend beyond general interpersonal skills to include communication tasks central to the OT process [1,4]. These include eliciting clients’ occupational histories, understanding values and life contexts, negotiating occupation-based goals, developing therapeutic relationships, and using the self intentionally in intervention [1,4]. Interprofessional and condition-specific communication education are important, but the findings indicate a need to make occupation-centered communication more visible in curricula and educational reports. Making these communication processes explicit may help learners connect communication skills with core OT practices, including collaborative goal setting, therapeutic relationship building, and client engagement in occupation-based intervention [1,4]. The mapped educational content may also inform continuing professional development planning by helping educators identify communication competencies that require reinforcement or further development after entry to practice.

Educational programs may also use the assessment approaches mapped in this review to select outcome measures that align with their communication learning objectives [76]. Reflective self-assessment can support learners’ awareness and confidence, but it may need to be complemented by objective or observer-rated approaches when the intended outcome is communication performance [76]. Examples include structured feedback, rubric-based ratings, standardized patient assessments, objective structured clinical examinations, and video-based review or interaction analysis of communication behavior [76,78,79].

### Implications for Future Research

Future research could examine whether communication education is associated with observable changes in communication behavior and whether these changes are sustained in clinical settings. Objective or observer-rated assessments, workplace-based evaluation, and longitudinal designs may help determine whether learners can apply communication skills beyond the educational setting [76,77]. Such studies should distinguish performance demonstrated in simulated or educational settings from communication applied in routine practice and should report the timing, clinical context, and raters used for assessment [77]. Further research is also needed on outcomes beyond the learning level. Future studies could examine whether communication education influences therapeutic relationship quality and client engagement and, where feasible, whether it is associated with occupational participation, quality of life, or broader practice-level outcomes. Evaluation approaches that combine multiple assessment methods and both quantitative and qualitative information may be useful for examining measurable behavioral change as well as learners’ experiences of applying communication skills in practice [79]. Postlicensure communication education remains underrepresented in the published literature and warrants further investigation. Communication demands may become more complex after entry to practice, as practitioners report challenges related to therapeutic use of self and therapeutic relationships [74], yet the published literature has focused mainly on prelicensure education. Future studies could examine continuing professional development programs for practicing occupational therapists and clarify how advanced communication competencies, such as therapeutic use of self and communication in complex clinical situations, can be taught and evaluated.

### Limitations

This review has some limitations. First, several aspects of the search process may have led to relevant sources being missed. The search was limited to English- and Japanese-language publications, the languages the review team could screen and chart reliably. In addition, 19 reports could not be retrieved for full-text assessment. Because the content and eligibility of these unavailable reports could not be assessed, their exclusion may have introduced availability-related selection bias, potentially affecting the mapped distribution of educational content, settings, and outcomes. Forward citation searching, searches of study registries, and contact with authors or experts were not undertaken because they were not prespecified in the review protocol and could not be completed within the review timeline. Formal PRESS review was considered during manuscript revision but could not be undertaken because an independent information specialist with appropriate expertise was not available within the revision period. Because backward citation searching identifies earlier references but not subsequent reports citing the included literature, the absence of forward citation searching may have resulted in relevant recent literature being missed. Furthermore, the included literature was concentrated in a small number of mainly English-speaking countries, and unpublished curricula and local continuing professional development programs may be underrepresented. This geographic concentration may limit the geographic and cultural representativeness of the mapped literature. Because exclusion decisions predominated, particularly during title and abstract screening, the κ estimates should be interpreted alongside percentage agreement. The resulting high expected chance agreement may have contributed to the discrepancy between the high percentage agreement and the moderate κ values. Second, although a review protocol was developed before the formal search and screening, it was not prospectively registered before the review began. The protocol was retrospectively archived on the Open Science Framework during manuscript revision [17], providing readers with access to the internal protocol. However, because public archiving occurred after completion of the review, it cannot independently verify that the methods were prespecified or clearly distinguish prospectively planned methods from decisions made during the review process. This limits the external verifiability of the review process. Third, no formal critical appraisal of individual sources was conducted because the purpose of this scoping review was to map the extent and characteristics of the existing literature rather than to evaluate the effectiveness of educational interventions. Consequently, the findings should be interpreted as a descriptive overview rather than evidence of educational effectiveness, and counts of assessment items and Kirkpatrick outcome levels should be interpreted as indicators of how outcomes were reported rather than as effect estimates. In addition, the boundaries of communication education were not always clearly defined in the included literature. Interprofessional education, teamwork, and client-centered practice sometimes incorporated communication-related elements without identifying communication as their primary educational focus. To improve conceptual consistency, we included only reports in which communication was explicitly described as an educational objective, educational content, or outcome assessment. Nevertheless, the included reports were heterogeneous in participants, educational content, delivery methods, and outcome measures, and some degree of interpretive judgment was unavoidable when determining eligibility and classifying educational content. Educational content and Kirkpatrick outcome levels were not independently coded by multiple reviewers. Therefore, interrater reliability was not assessed, and the classifications may have been influenced by reviewer judgment despite team review and consensus.

### Conclusions

This scoping review maps communication education across both prelicensure and postlicensure OT education in the English- and Japanese-language literature, extending previous reviews that examined specific educational methods such as simulation-based education [11] and coaching [12]. The findings indicate that, among the reports included in this review, most focused on prelicensure education and learner-reported outcomes; fewer addressed postlicensure education or observable communication behavior, and none assessed client- or practice-level outcomes. These findings can inform curriculum design, continuing professional development planning, and the selection of communication-related outcome measures in OT education. In particular, they suggest a need to make occupation-centered communication—such as eliciting occupational histories, negotiating occupation-based goals, developing therapeutic relationships, and applying the therapeutic use of self—more explicit in educational objectives and assessment. Future research could examine whether such education is associated with observable and sustained changes in communication behavior and with outcomes that are meaningful to clients and practice. The findings should be interpreted as a map of the published literature included in this review rather than as a comprehensive representation of communication education practices worldwide.

## Supplementary material

10.2196/95909 Multimedia Appendix 1 Search strategies.

10.2196/95909 Multimedia Appendix 2 Details of the included studies.

10.2196/95909 Checklist 1 PRISMA-ScR checklist.

## Abbreviations

OATD: Open Access Theses and Dissertations
OT: occupational therapy
PRESS: Peer Review of Electronic Search Strategies
PRISMA-S: Preferred Reporting Items for Systematic Reviews and Meta-Analyses Literature Search Extension
PRISMA-ScR: Preferred Reporting Items for Systematic Reviews and Meta-Analyses extension for Scoping Reviews
WFOT: World Federation of Occupational Therapists
