# Supplementary material for: Communication Education in Prelicensure and Postlicensure Occupational Therapy: Scoping Review
Source: JMIR Med Educ. 2026 Nov 13;12:e95909. doi: 10.2196/95909 (PMC13761669; doi:10.2196/95909)
Supplement: Supplementary file 1 [file mededu-v12-e95909-s001.docx]

**Multimedia Appendix 1. Search Strategies**

The search strategies were developed using three core concepts: occupational therapy, communication, and education. Controlled vocabulary terms were used where available, and free-text terms were adapted for each database or source. No publication year restrictions were applied.

**PubMed**

**Database/source:** PubMed
**Platform:** MEDLINE via PubMed
**Date searched:** May 25, 2026
**Limits/filters:** None
**Records:** 2,944

**Search concepts:**
1. Occupational therapy
2. Communication
3. Education

**Complete search string:**

**#1 Occupational therapy**

("Occupational Therapy"[Mesh] OR "Occupational Therapists"[Mesh] OR "occupational therap*"[tiab] OR "occupational therapist*"[tiab] OR "occupational therapy student*"[tiab] OR "occupational therapy education"[tiab] OR "occupational therapy curriculum"[tiab])

**#2 Communication terms**

("Communication"[Mesh] OR "Professional-Patient Relations"[Mesh] OR "Empathy"[Mesh] OR "Interview, Psychological"[Mesh] OR "Motivational Interviewing"[Mesh] OR communicat*[tiab] OR "communication skill*"[tiab] OR "clinical communication"[tiab] OR "patient communication"[tiab] OR "client communication"[tiab] OR "patient-centered communication"[tiab] OR "person-centered communication"[tiab] OR "client-centered communication"[tiab] OR interview*[tiab] OR counsel*[tiab] OR empathy[tiab] OR empathic[tiab] OR "active listening"[tiab] OR rapport[tiab] OR "interpersonal skill*"[tiab] OR "interpersonal relation*"[tiab] OR "therapeutic relationship*"[tiab] OR "therapeutic alliance"[tiab] OR "therapeutic use of self"[tiab] OR "motivational interviewing"[tiab] OR "shared decision making"[tiab] OR "interprofessional communication"[tiab] OR "team communication"[tiab])

**#3 Education terms**

("Education"[Mesh] OR "Education, Professional"[Mesh] OR "Education, Professional, Retraining"[Mesh] OR "Teaching"[Mesh] OR "Curriculum"[Mesh] OR "Simulation Training"[Mesh] OR "Patient Simulation"[Mesh] OR educat*[tiab] OR teach*[tiab] OR train*[tiab] OR learn*[tiab] OR curriculum*[tiab] OR course*[tiab] OR workshop*[tiab] OR simulation[tiab] OR "simulation-based"[tiab] OR "standardized patient*"[tiab] OR "standardised patient*"[tiab] OR "simulated patient*"[tiab] OR "role play*"[tiab] OR roleplay*[tiab] OR "e-learning"[tiab] OR "online learning"[tiab] OR "virtual patient*"[tiab] OR "virtual reality"[tiab] OR "continuing education"[tiab] OR "continuing professional development"[tiab] OR "professional development"[tiab] OR "clinical education"[tiab])

**#4 Combined search**

#1 AND #2 AND #3

**CINAHL**

**Database/source:** CINAHL
**Platform:** CINAHL
**Date searched:** May 25, 2026
**Limits/filters:** None
**Records:** 3,007

**Search concepts:**
1. Occupational therapy
2. Communication
3. Education

**Complete search string:**

**S1 Occupational therapy**

(MH "Occupational Therapy" OR MH "Occupational Therapists" OR TI "occupational therap*" OR AB "occupational therap*" OR TI "occupational therapist*" OR AB "occupational therapist*" OR TI "occupational therapy student*" OR AB "occupational therapy student*" OR TI "occupational therapy education" OR AB "occupational therapy education" OR TI "occupational therapy curriculum" OR AB "occupational therapy curriculum")

**S2 Communication**

(MH "Communication" OR MH "Professional-Patient Relations" OR MH "Empathy" OR MH "Interviewing" OR MH "Motivational Interviewing" OR TI communicat* OR AB communicat* OR TI "communication skill*" OR AB "communication skill*" OR TI "clinical communication" OR AB "clinical communication" OR TI "patient communication" OR AB "patient communication" OR TI "client communication" OR AB "client communication" OR TI "patient-centered communication" OR AB "patient-centered communication" OR TI "person-centered communication" OR AB "person-centered communication" OR TI "client-centered communication" OR AB "client-centered communication" OR TI interview* OR AB interview* OR TI counsel* OR AB counsel* OR TI empathy OR AB empathy OR TI empathic OR AB empathic OR TI "active listening" OR AB "active listening" OR TI rapport OR AB rapport OR TI "interpersonal skill*" OR AB "interpersonal skill*" OR TI "interpersonal relation*" OR AB "interpersonal relation*" OR TI "therapeutic relationship*" OR AB "therapeutic relationship*" OR TI "therapeutic alliance" OR AB "therapeutic alliance" OR TI "therapeutic use of self" OR AB "therapeutic use of self" OR TI "motivational interviewing" OR AB "motivational interviewing" OR TI "shared decision making" OR AB "shared decision making" OR TI "interprofessional communication" OR AB "interprofessional communication" OR TI "team communication" OR AB "team communication")

**S3 Education**

(MH "Education" OR MH "Education, Professional" OR MH "Teaching" OR MH "Curriculum" OR MH "Simulation Training" OR MH "Patient Simulation" OR TI educat* OR AB educat* OR TI teach* OR AB teach* OR TI train* OR AB train* OR TI learn* OR AB learn* OR TI curriculum* OR AB curriculum* OR TI course* OR AB course* OR TI workshop* OR AB workshop* OR TI simulation OR AB simulation OR TI "simulation-based" OR AB "simulation-based" OR TI "standardized patient*" OR AB "standardized patient*" OR TI "standardised patient*" OR AB "standardised patient*" OR TI "simulated patient*" OR AB "simulated patient*" OR TI "role play*" OR AB "role play*" OR TI roleplay* OR AB roleplay* OR TI "e-learning" OR AB "e-learning" OR TI "online learning" OR AB "online learning" OR TI "virtual patient*" OR AB "virtual patient*" OR TI "virtual reality" OR AB "virtual reality" OR TI "continuing education" OR AB "continuing education" OR TI "continuing professional development" OR AB "continuing professional development" OR TI "professional development" OR AB "professional development" OR TI "clinical education" OR AB "clinical education")

**S4 Combined search**

S1 AND S2 AND S3

**Web of Science**

**Database/source:** Web of Science
**Platform:** Web of Science
**Date searched:** May 25, 2026
**Limits/filters:** None
**Records:** 2,926

**Search concepts:**

1. Occupational therapy
2. Communication
3. Education

**Complete search string:**

**#1 Occupational therapy**

("occupational therap*" OR "occupational therapist*" OR "occupational therapy student*" OR "occupational therapy education" OR "occupational therapy curriculum")

**#2 Communication**

(communicat* OR "communication skill*" OR "clinical communication" OR "patient communication" OR "client communication" OR "patient-centered communication" OR "person-centered communication" OR "client-centered communication" OR interview* OR counsel* OR empathy OR empathic OR "active listening" OR rapport OR "interpersonal skill*" OR "interpersonal relation*" OR "therapeutic relationship*" OR "therapeutic alliance" OR "therapeutic use of self" OR "motivational interviewing" OR "shared decision making" OR "interprofessional communication" OR "team communication")

**#3 Education**

(educat* OR teach* OR train* OR learn* OR curriculum* OR course* OR workshop* OR simulation OR "simulation-based" OR "standardized patient*" OR "standardised patient*" OR "simulated patient*" OR "role play*" OR roleplay* OR "e-learning" OR "online learning" OR "virtual patient*" OR "virtual reality" OR "continuing education" OR "continuing professional development" OR "professional development" OR "clinical education")

**#4 Combined search**

#1 AND #2 AND #3

**ERIC**

**Database/source:** ERIC
**Platform:** ERIC
**Date searched:** May 25, 2026
**Limits/filters:** None
**Records:** 616

**Search concepts:**

1. Occupational therapy
2. Communication
3. Education

**Complete search string:**

**#1 Occupational therapy**

("occupational therapy" OR "occupational therapist" OR "occupational therapists" OR "occupational therapy student" OR "occupational therapy students" OR "occupational therapy education" OR "occupational therapy curriculum")

**#2 Communication**

(communication OR "communication skills" OR "clinical communication" OR "patient communication" OR "client communication" OR "patient-centered communication" OR "person-centered communication" OR "client-centered communication" OR interview OR interviewing OR counseling OR counselling OR empathy OR empathic OR "active listening" OR rapport OR "interpersonal skills" OR "interpersonal relations" OR "therapeutic relationship" OR "therapeutic relationships" OR "therapeutic alliance" OR "therapeutic use of self" OR "motivational interviewing" OR "shared decision making" OR "interprofessional communication" OR "team communication")

**#3 Education**

(education OR educational OR teaching OR training OR learning OR curriculum OR curricula OR course OR courses OR workshop OR workshops OR simulation OR "simulation based" OR "simulation-based" OR "standardized patient" OR "standardized patients" OR "standardised patient" OR "standardised patients" OR "simulated patient" OR "simulated patients" OR "role play" OR "role playing" OR roleplay OR roleplaying OR "e-learning" OR "online learning" OR "virtual patient" OR "virtual patients" OR "virtual learning" OR "virtual learning environment" OR "virtual reality" OR "continuing education" OR "continuing professional development" OR "professional development" OR "clinical education")

**#4 Combined search**

#1 AND #2 AND #3

**Ichushi-Web**

**Database/source:** Ichushi-Web
**Platform:** Japan Medical Abstracts Society
**Date searched:** May 25, 2026
**Limits/filters:** None
**Records:** 580

**Search concepts:**

1. Occupational therapy
2. Communication
3. Education

**Complete search string:**

**#1 Occupational therapy**

(作業療法 OR 作業療法士 OR 作業療法学生 OR occupational therapy OR occupational therapist*)

**#2 Communication**

(コミュニケーション OR 面接 OR 共感 OR 傾聴 OR 治療的関係 OR 治療的自己の活用 OR 多職種連携 OR communication OR communication skill* OR interview* OR empathy OR active listening OR therapeutic relationship OR therapeutic use of self OR motivational interviewing OR interprofessional communication)

**#3 Education**

(教育 OR 養成教育 OR 卒前教育 OR 卒後教育 OR 生涯教育 OR 研修 OR 講習 OR カリキュラム OR 学習 OR シミュレーション OR ロールプレイ OR education OR training OR curriculum OR learning OR simulation OR standardized patient* OR simulated patient* OR role play*)

**#4 Combined search**

#1 AND #2 AND #3

**Open Access Theses and Dissertations**

**Database/source:** Open Access Theses and Dissertations
**Platform:** OATD
**Date searched:** May 25, 2026
**Limits/filters:** None
**Records:** 170

#1 "occupational therapy"

#2 communication

#3 education

#4 #1 AND #2 AND #3
